# Characterization and Sequencing of a Genotype XII Newcastle Disease Virus Isolated from a Peacock (*Pavo cristatus*) in Peru

**DOI:** 10.1128/genomeA.00792-15

**Published:** 2015-07-30

**Authors:** Ana Chumbe, Ray Izquierdo-Lara, Luis Tataje-Lavanda, Aling Figueroa, Karen Segovia, Rosa Gonzalez, Giovana Cribillero, Angela Montalvan, Manolo Fernández-Díaz, Eliana Icochea

**Affiliations:** a Farvet S.A.C. Chincha Alta, Ica, Peru; b Laboratory of Avian Pathology, Facultad de Medicina Veterinaria, Universidad Nacional Mayor de San Marcos (UNMSM), Lima, Peru

## Abstract

Here, we report the first complete sequence and biological characterization of a Newcastle disease virus (NDV) isolated from a peacock in South America (NDV/peacock/Peru/2011). This isolate, classified as genotype XII in class II, highlights the need for increased surveillance of noncommercial avian species.

## GENOME ANNOUNCEMENT

Newcastle disease virus (NDV) is able to infect >250 species of birds worldwide and is also responsible for an acute and very contagious respiratory disease. NDV (genus *Avulavirus*, family *Paramixoviridae*) possesses a negative-sense single-stranded RNA genome. Wild birds have been implicated in the spread of the disease to domestic birds (1). Based on sequence analysis of the fusion protein (F) gene, NDV strains are classified in class I or II. Strains of class I are avirulent in chickens, whereas strains in class II can be lentogenic, mesogenic, or velogenic. Class II is subdivided into at least 18 genotypes from I to XVIII (2). Several outbreaks have been observed in Peru in recent years, but information regarding which genotypes are circulating is poor.

In 2011, 9 out of 10 peacocks (*Pavo cristatus*) died in a zoo farm in Huachipa, Lima, Peru, with nervous and respiratory symptoms. The virus isolation, from oral and cloacal swabs, was carried out in 9-day-old embryonated specific-pathogen-free (SPF) chicken eggs. Allantoic fluid (AF) was collected and assessed by hemagglutination and a hemagglutination inhibition test using NDV hyperimmune serum. Amplified AF was clarified, aliquoted, stored at −80°C, and quantified by plaque assay. The 50% lethal dose (LD_50_) was calculated in 4-week-old SPF chickens by 10-fold serial dilutions. The intracerebral pathogenicity index (ICPI) was calculated according to standard protocols (3). All protocols that included animals were approved by the Institutional Animal Care and Use Committee of the Universidad Nacional Mayor de San Marcos (UNMSM), Lima, Peru. The complete sequence of NDV/peacock/Peru/2011 was amplified by 8 pairs of overlapping oligonucleotide primers using the OneStep reverse transcription-PCR (RT-PCR) kit (Qiagen). Purified amplicons were cloned into the pGEM-T Easy (Promega) and sequenced with T7/SP6 primers. The assembly and alignment were made on SnapGene and Clustal W, respectively. Phylogenetic analysis and sequences comparison were done in MEGA 6.0.

Sequence analysis showed that the genome of NDV/peacock/Peru/2011 is 15,192 nucleotides (nt) long, with an insertion of 6 nt (CTCCAC) after position 1647 in the 5′ noncoding region of the nucleoprotein gene, compared with the LaSota strain (GenBank accession no. AF077761.1). Phylogenetic analysis of the complete coding region of the F gene placed the isolate within genotype XII in class II, together with the poultry/Peru/1918-03/2008 strain (GenBank accession no. JN800306.1), which was previously also isolated in Peru (4). A comparison of the complete genome sequences showed that the highest genetic identity (98.7%) was observed with poultry/Peru/1918-03/2008, while only 82.1% was observed with the LaSota strain.

A comparison between the LD_50_ (10^6.72^/ml) and the number of PFU revealed that at least 2 × 10^2^ PFU per chicken are required to kill 100% (5/5 chickens), and 20 PFU are able to kill 60% (3/5 chickens). The isolate showed an ICPI of 1.80, characteristic of velogenic NDV strains. These characteristics are consistent with the presence of a polybasic amino acid motif (^112^RRQKR↓F^117^) at the fusion protein cleavage site, which is considered a major molecular determinant of virulence for NDV (5).

The elucidation of the complete genome sequence of NDV/peacock/Peru/2011 will help us further study the epidemiology and molecular pathogenesis of genotype XII and aid in the development of a vaccine candidate against homologous genotypes that are circulating in Peru.

### Nucleotide sequence accession number.

The complete genome sequence of NDV/peacock/Peru/2011 has been deposited in GenBank under the accession no. KR732614. The version described in this paper is the first version.
